# Identification of new candidate biomarkers to support doxorubicin treatments in canine cancer patients

**DOI:** 10.1186/s12917-021-03062-x

**Published:** 2021-12-07

**Authors:** Kristine Walters, Alessia Stornetta, Foster Jacobs, Peter W. Villalta, Maria Razzoli, Marianne Grant, Beshay Zordoky, Alessandro Bartolomucci, Antonella Borgatti, Silvia Balbo

**Affiliations:** 1 Department of Veterinary Clinical Sciences, College of Veterinary Medicine, University of Minnesota, 1365 Gortner Avenue, St Paul, MN 55108 USA; 2 WestVet 24/7 Animal Emergency & Specialty Center, 5024 W Chinden Boulevard, Garden City, ID 83714 USA; 3 Masonic Cancer Center, University of Minnesota, 2231 6th Street Southeast, Minneapolis, MN 55455 USA; 4 Division of Environmental Health Sciences, School of Public Health, University of Minnesota, 420 Delaware Street SE, Minneapolis, MN 55455 USA; 5 Department of Integrative Biology and Physiology, University of Minnesota, 2231 6th Street SE, Minneapolis, MN 55455 USA; 6 Department of Experimental and Clinical Pharmacology, College of Pharmacy, University of Minnesota, 308 Harvard Street S.E, Minneapolis, MN 55455 USA; 7 Clinical Investigation Center, College of Veterinary Medicine, St. Paul, MN 55108 USA

**Keywords:** DNA adducts, Adductomics, Doxorubicin, Predictive biomarker, Precision medicine, Veterinary oncology

## Abstract

**Background:**

Both human and veterinary cancer chemotherapy are undergoing a paradigm shift from a “one size fits all” approach to more personalized, patient-oriented treatment strategies. Personalized chemotherapy is dependent on the identification and validation of biomarkers that can predict treatment outcome and/or risk of toxicity. Many cytotoxic chemotherapy agents, including doxorubicin, base their mechanism of action by interaction with DNA and disruption of normal cellular processes. We developed a high-resolution/accurate-mass liquid chromatography-mass spectrometry DNA screening approach for monitoring doxorubicin-induced DNA modifications (adducts) in vitro and in vivo. We used, for the first time, a new strategy involving the use of isotope-labeled DNA, which greatly facilitates adduct discovery. The overall goal of this work was to identify doxorubicin-DNA adducts to be used as biomarkers to predict drug efficacy for use in veterinary oncology.

**Results:**

We used our novel mass spectrometry approach to screen for adducts in purified DNA exposed to doxorubicin. This initial in vitro screening identified nine potential doxorubicin-DNA adduct masses, as well as an intense signal corresponding to DNA-intercalated doxorubicin. Two of the adduct masses, together with doxorubicin and its metabolite doxorubicinol, were subsequently detected in vivo in liver DNA extracted from mice exposed to doxorubicin. Finally, the presence of these adducts and analytes was explored in the DNA isolated from dogs undergoing treatment with doxorubicin. The previously identified nine DOX-DNA adducts were not detected in these preliminary three samples collected seven days post-treatment, however intercalated doxorubicin and doxorubicinol were detected.

**Conclusions:**

This work sets the stage for future evaluation of doxorubicin-DNA adducts and doxorubicin-related molecules as candidate biomarkers to personalize chemotherapy protocols for canine cancer patients. It demonstrates our ability to combine in one method the analysis of DNA adducts and DNA-intercalated doxorubicin and doxorubicinol. The last two analytes interestingly, were persistent in samples from canine patients undergoing doxorubicin chemotherapy seven days after treatment. The presence of doxorubicin in all samples suggests a role for it as a promising biomarker for use in veterinary chemotherapy. Future studies will involve the analysis of more samples from canine cancer patients to elucidate optimal timepoints for monitoring intercalated doxorubicin and doxorubicin-DNA adducts and the correlation of these markers with therapy outcome.

**Supplementary Information:**

The online version contains supplementary material available at 10.1186/s12917-021-03062-x.

## Background

Traditionally, cancer has been treated as a homogenous disease with chemotherapeutic treatment decisions based on tumor location, histopathologic findings, and expected biologic behavior [1]. However, genetic variations in patients can result in different responses to therapy and varying degrees of toxicity, despite phenotypically similar diseases [2, 3]. For these reasons, cancer chemotherapy is currently shifting from the concept of “one size fits all” to more personalized, patient-oriented approaches, with the goal of optimizing individual therapeutic protocols to increase treatment success and/or decrease undesired side effects [1].

Personalized chemotherapy is based on the ability to identify and target a patient subpopulation, predict drug efficacy, patient response, and likelihood of toxicity. The identification and validation of predictive biomarkers, robust chemical or molecular indicators of the outcome selected, is essential for identifying those patients who will most likely benefit from a drug regimen or will need a dose modification from the standard dosage [4, 5]. For example, a drug dose or a combination drug protocol may be adapted as a result of biomarker measurement to allow for less unwanted side effects without compromising treatment success.

There are multiple reports of identification and use of predictive biomarkers with traditional chemotherapeutics in a variety of human cancer types including, but not limited to, colorectal, breast, pancreatic and lung cancers [6–9]. Clinically, however, biomarkers are most commonly used to select patients for treatment with targeted therapies including monoclonal antibodies and small molecule inhibitors, but have not yet been implemented to guide treatment with traditional cytotoxic chemotherapy [10–12].

Patient-oriented treatment approaches have recently become of interest for use with veterinary patients, where the treatment goal is to provide a good quality of life while extending patient survival [13]. In veterinary medicine, there is sparse information regarding biomarker development and use, and there are no predictive biomarkers used routinely. Similarly, the use of personalized chemotherapy in veterinary patients is limited, the closest example of which is the use of a receptor tyrosine kinase inhibitor, toceranib (Palladia), for treatment of canine cutaneous mast cell tumors (cMCTs) [14]. Palladia works, in part, by inhibiting the receptor tyrosine kinase KIT resulting in an antiproliferative effect in cancer cells [15]. A large minority of canine cMCTs possess a mutation in the *c-kit* gene, and in one study, cMCTs with activating mutations in the *c-kit* gene were approximately twice as likely to respond to treatment with toceranib than those with wildtype *c-kit* [14].

Biomarker development and application of personalized chemotherapy approaches in veterinary medicine is of particular interest in guiding the practice of dose escalation of routinely used chemotherapeutic drugs [16, 17]. By identifying predictive biomarkers of patient response, dose escalation strategies can be modified for each individual to benefit both those who are more likely to respond to the drug used and those who are likely to have a poor response or higher risk of treatment-associated side effects. One example of clinically used dosing strategies to minimize risk of treatment-associated side effects is in treatment of dogs with mutations in the *ABCB1* (*MDR1*) gene. This gene encodes for the drug efflux pump, p-glycoprotein, dysfunction of which can lead to severe adverse drug reactions to many commonly used medications, including multiple chemotherapeutics, due to increased central nervous system exposure to the drug [18]. There is not a dosing strategy proven to be effective in decreasing this risk for dogs with *MDR1* gene mutations, and therefore, either a dose reduction of the chemotherapy drug or choosing an alternate chemotherapeutic that is not a substrate for p-glycoprotein is recommended [18]. Research has investigated the pharmacokinetics of chemotherapeutics in relation to the risk of myelotoxicity [19, 20], but these strategies have not been clinically adopted for use in personalized veterinary chemotherapy.

**Fig. 1:**
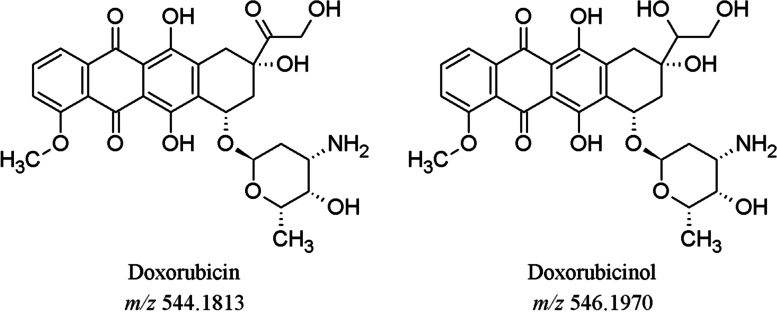
Doxorubicin and Doxorubicinol

Doxorubicin (DOX, Fig. 1), a member of the anthracycline group of compounds, has good anticancer activity against a wide spectrum of tumors including hematopoietic neoplasia, sarcomas, and carcinomas.

It is currently one of the most extensively used chemotherapeutic drugs in canine clinical settings [21, 22]. Treatment with DOX is not universally effective and may lead to adverse events, including dose-dependent cardiotoxicity. The intensity of these adverse events varies from patient to patient [21, 22]. Given its extensive use in cancer therapy, the development of predictive biomarkers is of particular relevance for management of DOX chemotherapy. The key component of the mechanism of action (MOA) of DOX is the poisoning of topoisomerase II through intercalation into DNA, but other cellular responses have been shown to contribute to its MOA, including the formation of DNA modifications (adducts) [23, 24].

DNA adducts from anticancer DNA alkylating drugs have been shown to be good candidate predictive biomarkers of drug efficacy [25]. Monitoring these adducts as predictive biomarkers has the advantage of providing an integrative measure of patient-specific responses, since they account for an individual’s absorption, distribution, metabolism, elimination and DNA repair [25]. Furthermore, drug-DNA adducts may be more suitable biomarkers, as compared to non-drug related metabolites because of their specificity [25]. The direct interaction of DOX with DNA creates an excellent opportunity for evaluating DOX-DNA adducts as predictive biomarkers. Previous in vitro studies have characterized a single DOX-DNA adduct generated in the presence of formaldehyde [26, 27], but to our knowledge, this or any other DOX-DNA adducts have yet to be detected in vivo.

Detection of DNA adducts in chemotherapy patients can be especially challenging because adducts develop at low levels beyond the typical detection limits achieved by traditional low-resolution spectral detection and high analytical flow rates liquid chromatography-mass spectrometry (LC-MS) methods [28, 29]. We previously developed a nanoLC-MS^3^ DNA adductomics approach that allows for the screening of potentially every adduct in a hydrolyzed DNA sample. This method is based on high-resolution/accurate-mass (HRAM) data-dependent constant neutral loss monitoring of the 2′-deoxyribose or one of the four DNA bases (guanine (G), adenine (A), thymine (T), and cytosine (C) [30, 31]. The accurate mass measurement of an observed DNA adduct can be used for determining its elemental composition, whereas the triggered MS^2^ and MS^3^ fragmentation spectra provide structural information of the modified base. In addition, the use of nanoflow (300 nL/min) and nanoelectrospray increases sensitivity by providing increased ionization and sampling efficiency [30, 31]. The goals of our study were to optimize our adductomics approach to screen for DOX-DNA adducts in vitro and in vivo and to identify candidate predictive biomarkers of DOX efficacy for future investigation in clinical studies.

## Results

### Screening for DOX-DNA adducts in vitro

**Fig. 2:**
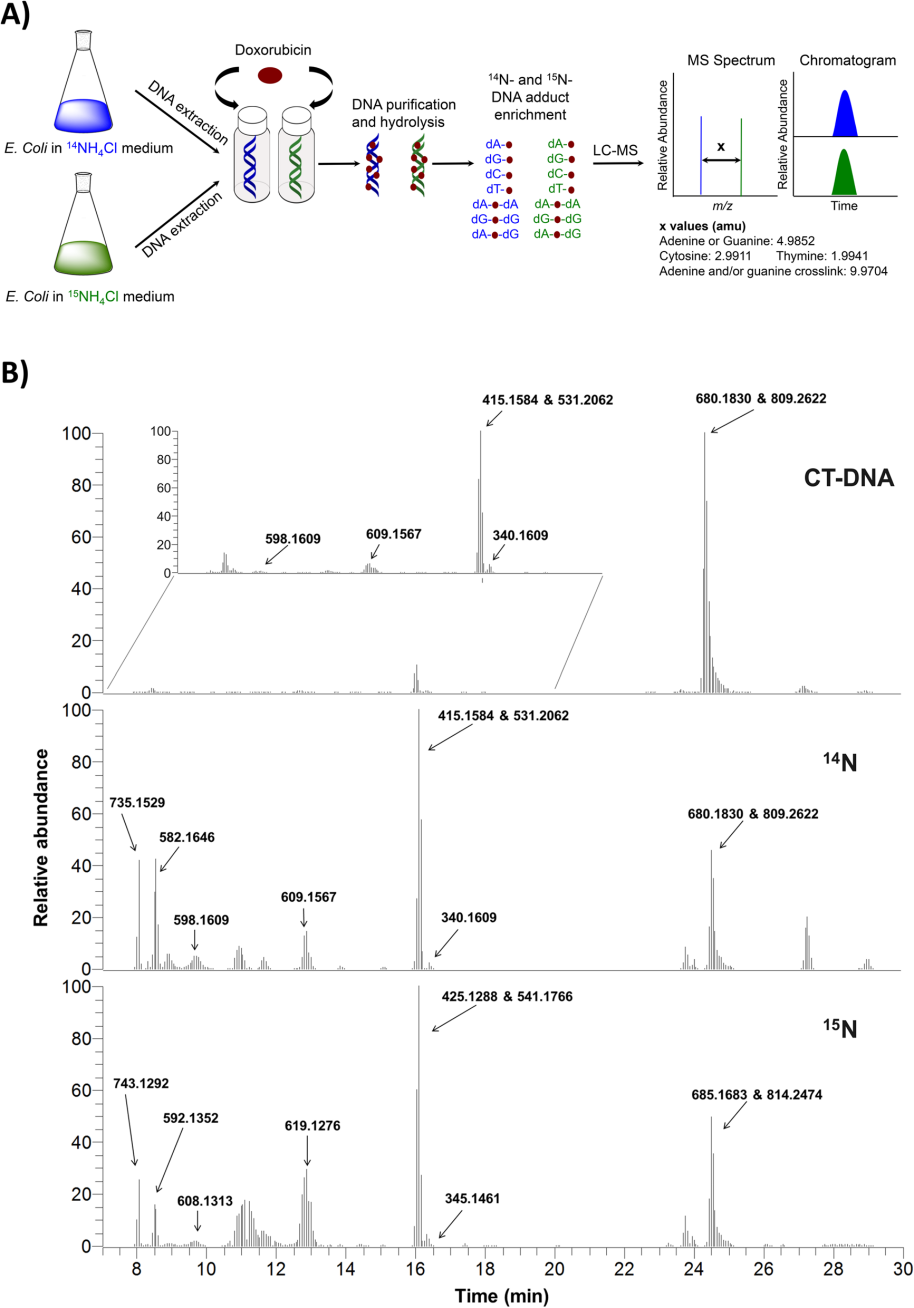
**A** Generation of ^15^N-labeled bacterial DNA and concept of mass pairing to facilitate adduct detection. Values correspond to mass differences (^14^N- in blue and ^15^N- in green) for each DNA base depending on the number of nitrogens present. **B** Extracted ion chromatograms (EIC, 5 ppm) of the exact masses that triggered an MS^3^ fragmentation event from the reaction of DOX with CT-DNA (top), ^14^N-bacterial DNA (center), or ^15^N-bacterial DNA (bottom) in the presence of formaldehyde. Only those masses that triggered an MS^3^ in DOX-treated samples, but not in the negative controls are reported

Initial screening for DOX-DNA adducts was performed by reacting DOX in the presence of formaldehyde with DNA from calf thymus (CT-DNA) and with DNA extracted from *E.coli* bacteria. In order to facilitate adduct detection, we implemented a new strategy based on the use of ^15^N-isotope-labeled DNA, generated in *E.coli* bacteria, to be paired with ^14^N unlabeled *E.coli* bacterial DNA. Both DNA species are subjected to the same DOX exposure and sample preparation protocols, and then the two samples (^14^N- and its counterpart ^15^N-bacterial DNA) are combined in a 1:1 ratio prior to LC-MS analysis. In this resulting combined sample, DNA adduct detection is based on the selection of only masses that triggered an MS^3^ fragmentation event in the drug-exposed DNA samples, and were present as a matching pair of ^14^N-DNA and ^15^N-DNA adducts, resulting in co-eluting peaks when extracted in the full scan chromatogram (this concept is explained in Fig. 2A).

A total of nine DNA adduct masses was detected in CT-DNA and ^14^N- and ^15^N-bacterial DNA exposed to DOX (Table 1 and Fig. 2B). None of these adducts were detected in the untreated controls.

**Table 1:** DOX-DNA adduct masses detected by untargeted screening from reaction of DOX with purified DNA in the presence of formaldehyde. Only the masses that triggered an MS^3^ fragmentation event in the DOX-exposed samples, but not in the negative control samples (unreacted DNA and buffer and enzymes used for the DNA hydrolysis) are reported. dR: 2′-deoxyribose, A: adenine, G: guanine, C: cytosine

No.	Parent Mass (***m/z***)	Product Mass (***m/z***)	Neutral Loss	Proposed Chemical Formula	CT-DNA	^**14**^N- and ^**15**^N-DNA Coupling
1	340.1609	224.1124	dR	C_12_H_20_O_4_N_8_	x	x
2	415.1584	264.1085	G	C_16_H_19_O_4_N_10_	x	x
		299.1103	dR		x	x
3	531.2062	415.1577	dR	C_21_H_27_O_7_N_10_	x	x
4	582.1646	447.1082	A	C_5_H_31_O_10_N_17_P_3_	x	x
5	598.1609	447.1110	G	C_15_H_27_O_12_N_12_P		x
6	609.1567	458.1090	G	C_24_H_17_O_5_N_16_	x	x
7	680.1830	564.1360	dR	C_27_H_38_O_6_N_7_P_4_	x	x
8	735.1529	624.1096	C	C_28_H_35_O_8_N_8_P_4_		x
9	809.2622	693.2137	dR	C_26_H_39_O_9_N_18_P_2_	x	x

These masses were detected upon neutral loss of dR, G, A or C. The most frequent neutral loss observed was dR, followed by G, observed five and three times, respectively. Overall, the majority of the adducts were evenly distributed over the 44-minute long chromatographic gradient (Fig. 2B). Two pairs of masses, however, eluted at the same retention time (RT, in Table 1 No. 2 and 3, and No. 7 and 9), suggesting that the pair belongs to the same molecule, and the lower mass is most likely the product of in-source fragmentation of the higher mass in the mass spectrometer.

In the first pair of masses, *m/z* 531.2062, was detected by neutral loss of dR resulting in a fragment ion of mass *m/z* 415.1577, which in turn triggered two MS^3^ fragmentation events upon neutral loss of guanine and dR, suggesting that this adduct is a crosslink comprising two dR moieties. Indeed, masses *m/z* 531.2062 and 415.1577 were assigned to a previously detected crosslink formed by a deoxyguanosine, formaldehyde, and deoxyadenosine (dG-CH_2_-dA) [32].

**Fig. 3:**
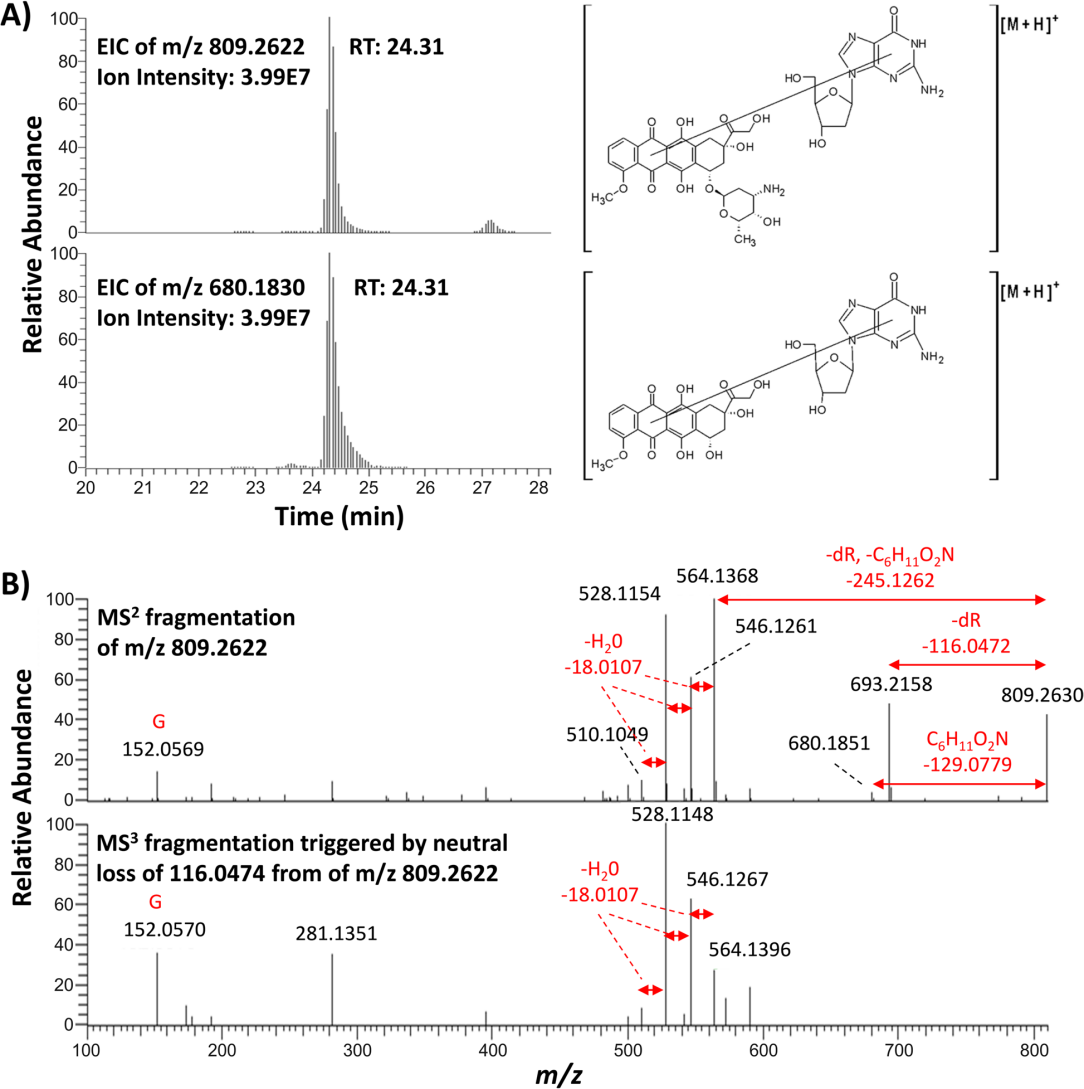
**A** EIC of *m/z* 809.2622 and *m/z* 680.1830. **B** MS^2^ and MS^3^ fragmentation spectra of *m/z* 809

The second pair of masses, *m/z* 809.2622 and 680.1830 (Fig. 3A) differed by 129.0792 amu, which corresponds to the exact mass of the aminosugar of DOX (Fig. 1).

 The data supports that *m/z* 680.1830 results from in-source fragmentation of the aminosugar from *m/z* 809.2622. Interpretation of the resulting MS^2^ and MS^3^ spectra suggests that this is a nucleoside adduct involving guanine, and that the aminosugar moiety of DOX, which is partly lost in the MS source, is not the moiety that reacts with DNA, as reported previously (Fig. 3B) [23]. Literature reports that the reduction of the quinone to a semiquinone results in a radical that adds to either the C4-, C5-, C8- or, to a much lesser extent, to the C2-position of guanine [33]. However, our in vitro system had no metabolic capacity and therefore these masses could originate from decomposition products of the drug. The chemical synthesis and characterization, and the matching of identical fragmentation spectra, is necessary for unequivocal adduct identification and will be the focus of future studies.

### Time course of formation and persistence of DOX-DNA adducts in vivo

The presence of the DOX-DNA adducts detected in vitro was then investigated in vivo using a targeted MS/MS approach in DNA extracted from liver samples harvested from mice exposed to two different DOX regimens and followed over time. In the first regimen, mice were acutely exposed to DOX, whereas in the second regimen, mice received a low dose of DOX once a week for 3 weeks. The samples from these studies were used to assess the kinetics of formation of the DOX-DNA adducts and their persistence over time, considering various time points after drug administration.

**Fig. 4:**
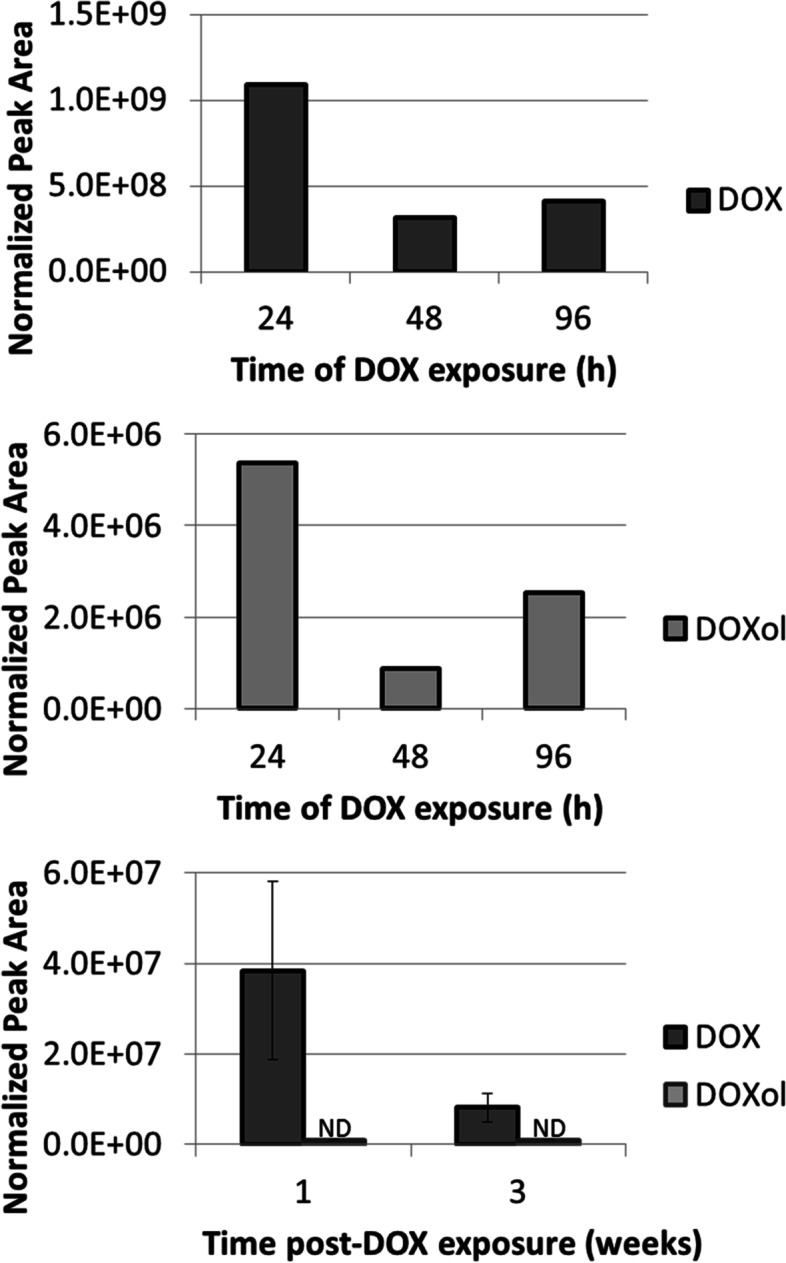
Detection of DOX and DOXol and persistence in DNA extracted from the liver of mice. DOX and DOXol were detected in one mouse treated acutely with 10 mg/kg of DOX (top and center plot, *n* = 1). Only DOX was detected in DNA from mice (*n* = 3) treated chronically with 4 mg/kg/week of DOX for three weeks (bottom plot). Peak areas were normalized by DNA amounts (derived by measuring amounts of dG). ND, not detected. Error bars represent the standard error of the mean for three biological replicates

In addition to the DOX-DNA adducts, our in vitro screening of hydrolyzed DNA samples revealed the presence of a very intense full scan peak with *m/z* 544.1813. This mass corresponds to the molecular ion of DOX (calculated *m/z* of 544.1813), suggesting that DOX is still intercalated in the DNA after sample purification using chloroform/isoamyl alcohol. In light of this finding, our targeted MS/MS approach also included the masses of DOX and, to account for metabolism, DOX’s major metabolite doxorubicinol (DOXol, *m/z* 546.1970, Fig. 1) [21]. DOX was detected in hydrolyzed DNA extracted from the liver of mice exposed for 24, 48, and 96 h, whereas DOXol was also detected in all three samples, but at an intensity that was about 150-to-350-fold lower than DOX, assuming similar ionization efficiency and recovery (Fig. 4).

Interestingly, DOX was still present in hydrolyzed DNA isolated from mouse liver one and three weeks post-drug exposure (Fig. 4), and at levels that were more than 2000-times above our limit of detection (LOD) of 33.3 fmol on-column (measured by triplicate injection of decreasing concentrations of DOX in matrix).

Due to the presence of DNA-intercalated DOX and DOXol in these samples, we considered the possibility that leftover DNA-intercalated drug is reacting with DNA bases during hydrolysis and sample cleanup, resulting in adduct formation in situ. We first attempted to remove the intercalated DOX from the DNA by performing 5 or 10 liquid/liquid extractions with phenol/chloroform/isoamyl alcohol, but this was shown not to be sufficient at removing the drug from the DNA (data not shown). Subsequently, ^15^N-labeled DNA in amounts equal to what was extracted from the mouse livers was added to the samples prior to DNA hydrolysis to check for adduct formation during sample preparation.

**Fig. 5:**
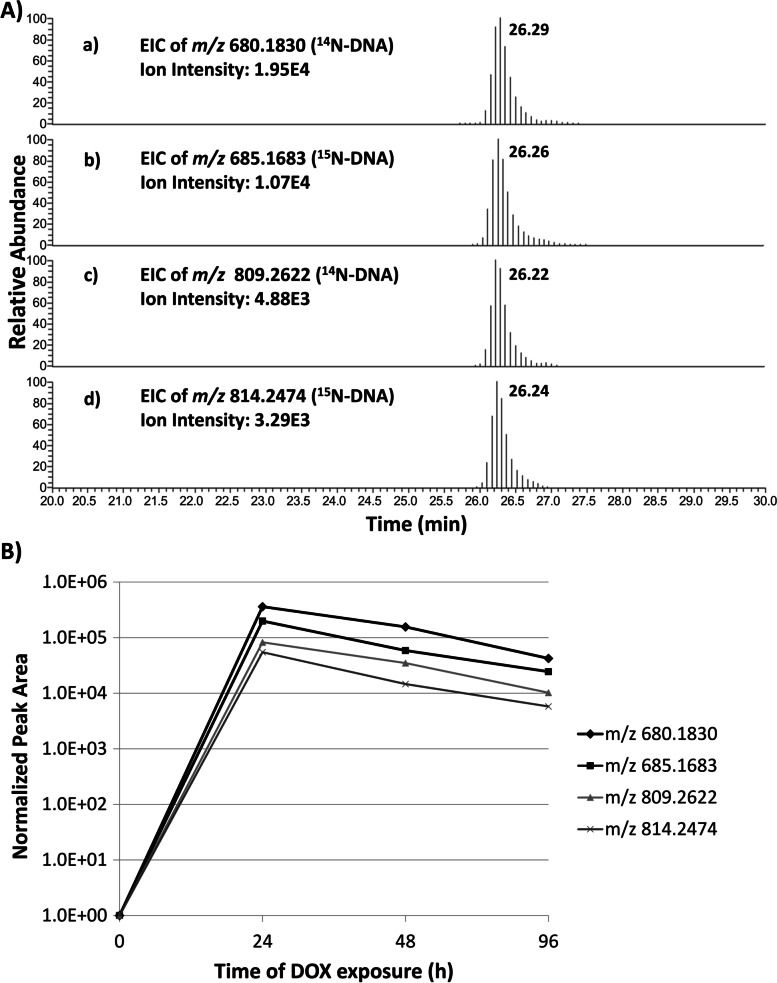
**A** Representative EIC of DOX-DNA adduct masses detected in mouse liver DNA (24 h DOX exposure). Chromatograms a and c correspond to masses found as ^14^N-DNA (mouse liver DNA), whereas b and d correspond to the same masses found as ^15^N-DNA (bacterial DNA spiked into the sample prior to processing). **B** Time course of DOX-DNA adduct formation for *m/z* 680.1830, 809.2622 and their ^15^N-labeled counterparts (*m/z* 685.1683 and 814.2474). Peak areas were normalized by DNA amounts (derived by measuring amounts of dG)

Two of the previously detected DOX-DNA adduct masses (*m/z* 680.1830 and 809.2622 in Table 1) were detected in liver DNA of mice exposed to DOX (Fig. 5A, plot a and c).

Interestingly, their ^15^N-labeled counterparts were also present, suggesting that these two masses are also formed during sample processing (Fig. 5A, plot b and d). The time course of formation over 96 h exposure of these two masses (and their ^15^N-labeled counterparts) showed similar levels and trends for each drug exposure duration, suggesting that this adduct is mostly formed during sample processing rather than in vivo during drug exposure (Fig. 5B). These two DOX-DNA adduct masses were not detected in the liver DNA samples of mice after one or three weeks from treatment with DOX.

### Detection of DOX-adducts, DOX and DOXol in DNA isolated from blood of canine cancer patients

Previously detected DOX-DNA adducts (Table 1), DOX and DOXol were targeted (MS/MS) for detection in DNA isolated from three canine patients receiving DOX as part of a multiagent chemotherapy protocol called CHOP (Cyclophosphamide, Hydroxydaunorubicin (DOX), Oncovin (vincristine), and Prednisone). A single blood sample (about 3 mL) was collected from each canine patient one week post-treatment with DOX, when dogs returned to the clinic for a post-chemotherapy complete blood count (CBC) per routine protocol at the hospital (Table 2).

**Table 2:** Canine cancer patient information

	Dog 1	Dog 2	Dog 3
**Signalment**	12 year old male neutered German Shepherd Dog	6 year old female spayed Irish Setter	9 year old male neutered Cocker Spaniel
**Cancer type**	High grade lymphoma	High grade lymphoma	High grade lymphoma
**Chemotherapy protocol**	Modified UW-Madison CHOP-19	UW-Madison CHOP-19	UW-Madison CHOP-19
**Number of doxorubicin treatments at sample collection**	5	3	4
**Doxorubicin dose (mg/m**^**2**^**)**	29.6	29.9	29.8
**Doxorubicin dose (mg/kg)**	0.9	0.9	1.4
**Absolute blood neutrophil count at time of sample collection; reference range 2.10–11.2 × 10**^**3**^**/ul**	2.12 × 10^3^/ul	2.88 × 10^3^/ul	5.25 × 10^3^

**Fig. 6:**
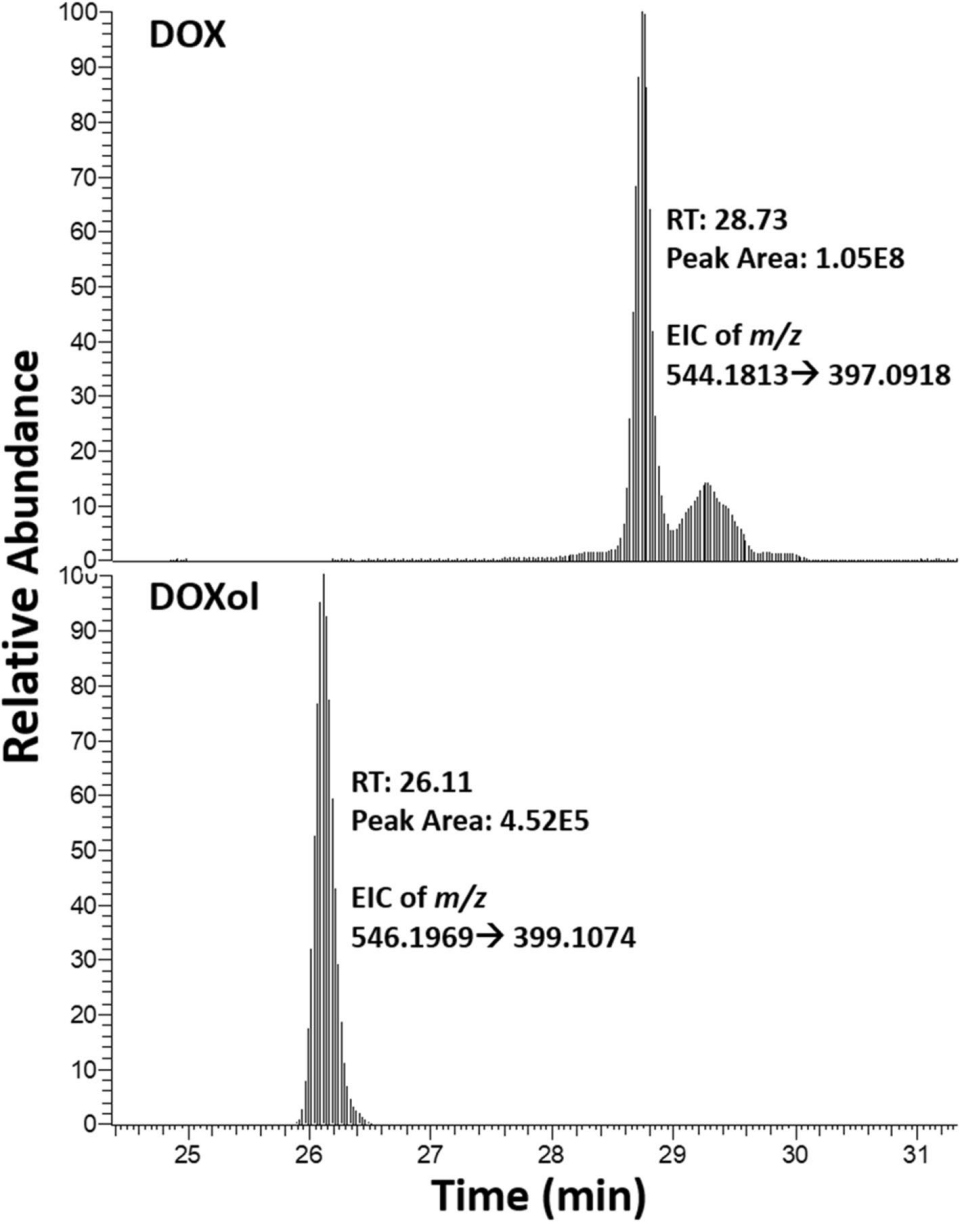
Targeted MS^2^ of DOX and DOXol in DNA isolated from blood collected from a canine cancer patient (Dog 3) one week after receiving DOX

Blood samples collected from two dogs who did not receive DOX were used as a negative control. Extracted DNA amounts ranged from 90 to 200 μg. None of the previously observed DOX-DNA adducts were detected in the samples. DOX was detected in the DNA isolated from all three exposed dog blood samples, whereas DOXol was detected in the DNA of two out of three samples. Figure 6 is a typical example of the extracted ion chromatograms for DOX and DOXol in canine patients.

## Discussion

In this study, we applied our LC-MS^3^ adductomics approach to screen for DNA adducts induced by the anticancer drug doxorubicin (DOX) both in vitro and in vivo. The main findings of this study are 1) a novel LC-MS^3^-based approach that detects DOX-DNA adducts, DOX, and DOXol; 2) a list of DOX-DNA adduct masses detected in vitro and in vivo; 3) information about the persistence over time of DNA-intercalated DOX and doxorubicinol, in mice receiving DOX and canine cancer patients undergoing DOX treatment; and 4) identification of promising analytes to be developed as predictive biomarkers to support DOX treatment and to be validated for future use in veterinary oncology.

In cancer chemotherapy, precision medicine-based approaches using biomarkers of efficacy are being developed to predict a patient’s response to the treatment. Previous studies have shown promise for the use of predictive biomarkers as an alternative to more conventional dose-determining methods. However, in veterinary medicine, there are limited examples that have been demonstrated, but are not commonly used clinically. For example in cats undergoing chemotherapy, a biomarker-based personalized approach for treatment with carboplatin better predicted myelosuppression than dosing based on body surface area [20]. The serum concentration-time curve for DOX has also been found to be predictive of the reduction of total white blood cell and neutrophil counts in dogs [19].

In the case of DNA adducts as predictive biomarkers, various studies have investigated in humans the relationship between DNA adducts and patient treatment outcome [25]. One study found that by measurement of the interstrand DNA cross-link G-NOR-G, it was determined that Fanconi anemia (FA) patients are hypersensitive to the anticancer drug cyclophosphamide and require a lower dose of the drug compared to non-FA patients prior to hematopoietic cell transplantations [34]. Another study found that out of seven patients being treated for multiple myeloma, the three with the lowest levels of DNA adducts in *TP53* and *N-ras* gene sequences did not respond to treatment with melphalan [35].

With regards to platinum-based chemotherapy, higher levels of platinum-DNA adducts have been observed in isolated leukocyte DNA in patients with good clinical outcome when being treated for ovarian and testicular cancer with cisplatin or carboplatin [36–38]. Platinum-DNA adduct formation has also been found to correlate significantly with patient response following treatment for non-small-cell lung cancer with cisplatin [39]. In a study that investigated Oxaliplatin, it was observed that platinum-DNA adduct levels in peripheral blood mononuclear cells correlated significantly to mean tumor volume change [40]. Finally, carboplatin-DNA adduct levels following diagnostic microdoses have been investigated for their potential to predict patient response prior to treatment with the therapeutic dose [41].

Our in vitro screening approach, which resulted in the detection of nine DOX-DNA adduct masses (Table 1), was improved by using a novel strategy involving the pairing of ^14^N- and ^15^N-labeled DNA (Fig. 2A). This novel strategy facilitates adduct detection and can be applied for in vitro identification of DNA adducts resulting from any type of exogenous exposure, such as other DNA alkylating anticancer drugs, as well as tobacco-specific, dietary, and environmental chemicals.

Early studies reported the detection, in vitro*,* of a DNA adduct formed at the N^2^-position of guanine that involved formaldehyde to link the DNA to the aminosugar of DOX [23]. This adduct was not detected in vitro by our approach. We hypothesize that the previously reported poor stability of this adduct in DNA [23] makes its detection, in hydrolyzed samples and after using our approach and current conditions, challenging. In an effort to make this adduct more stable, we performed a reduction using sodium cyanoborohydride [42], however the adduct was not detected in its reduced form (data not shown).

Additionally, an interesting finding from our in vivo adduct detection was the formation of adducts during sample preparation. We hypothesize that the release of DOX in the solution, as a consequence of the DNA being hydrolyzed, results in its reaction with free nucleosides to form DNA adducts. To our knowledge, there is no information currently available about the reactivity of DOX with free nucleosides, suggesting that the nature of this reaction as well as the persistence of DNA-intercalated DOX needs further characterization. If adduct formation is greater when DOX is released from the DNA (such as during enzyme hydrolysis), it is possible that in vivo adduct formation takes place in the course of DNA replication, during which the double helix opens up to allow for the synthesis of a new DNA strand and the intercalated DOX is released. Furthermore, adduct formation during sample processing seems to be solely a characteristic of those drugs that are able to intercalate to DNA, but not of drugs whose structure does not allow for such intercalation. Indeed, a complete removal from the DNA of the anticancer drug cyclophosphamide is possible when using similar sample preparation protocols and in situ formation of adducts during DNA hydrolysis is not observed (data not shown). Understanding if this is a feature of all drugs or molecules that intercalate to DNA will be the focus of future work.

To verify the presence of the DOX-DNA adducts, DOX, and DOXol in a sample type that would be available for biomarker monitoring in the clinic, we analyzed DNA isolated from blood collected from dogs undergoing chemotherapy treatment that included DOX (seven days post-treatment). Because none of the previously observed adducts were detected in these samples, we hypothesize that too much time has passed between sample collection and treatment, and therefore levels of adducts were most likely below the limit of detection of our approach. On the other hand, DOX and DOXol were detected in the DNA extracted from these samples (Fig. 6). The ability of our approach to measure DOX in DNA from patient samples using as little as 3 mL of blood demonstrates the feasibility of using intercalated DOX as a potential predictive biomarker of efficacy. A different study reported an assay for quantification of DOX intercalated with DNA in tumor and tissues using HPLC [43]. In comparison, our LC-MS DNA adductomics approach has the advantage of providing a combined measurement of DOX-DNA adducts, DOX and DOXol, as well as structural information through fragmentation spectra, which can be used to confirm the structure of anticipated molecules, identify the structures of new ones, and facilitate peak assignment in the absence of an isotope-labeled internal standard.

## Conclusions

The adoption of personalized approaches in veterinary oncology has the potential not only for increased treatment success, but also to be more cost-effective as cancer chemotherapy for animals can be expensive. Our study provides new insights on promising potential DNA markers to be developed as predictive tools in canine cancer treatment with DOX. To our knowledge, this is the first study that uses a DNA adductomics screening approach for the combined analysis of a clinically used drug and its derived DNA adducts. We demonstrated the ability of our method to monitor DOX in DNA isolated from blood collected from canine cancer patients seven days post-treatment, suggesting that DNA-intercalated DOX may be developed as a predictive biomarker of drug efficacy. Future efforts will focus on measuring intercalated DOX to select veterinary patients that will benefit from chemotherapy and to develop personalized chemotherapy protocols aimed at improving quality of life of canine cancer patients.

## Methods

### Reagents and chemicals

Cell lysis, Proteinase K, and RNase A solutions were purchased from QIAGEN. DNA purified from calf thymus (CT-DNA) was purchased from Worthington Biochemical Corporation, C_3_H_8_O and CH_3_OH were purchased from Honeywell, and CH_2_O (37%), MgSO_4_, and CaCl_2_ were purchased from Thermo Fisher Scientific. All other chemicals, materials and enzymes were purchased from Millipore Sigma. All solvents used for chromatography and mass spectrometry analyses were of the purest commercially available grade.

### Generation of isotope-labeled DNA from *E.coli*

^15^N-labeled bacterial DNA was generated by growing *E.coli* (MG1655 strain) in M9 minimal medium (standard) fortified with ^15^NH_4_Cl. 98% DNA labeling was achieved by growing the bacteria for at least three generations. Briefly, 10 μL of bacterial stock culture in 25% glycerol were inoculated in 5 mL M9 minimal media starter culture and incubated overnight in a thermoshaker (37 °C, 200 rpm). Afterwards, 50 μL of cells from the starting culture were added to 1 L M9 minimal medium containing ^15^NH_4_Cl and further incubated in the thermoshaker (37 °C, 200 rpm) until an optical density (measured by absorbance at 600 nm) of 1.2 absorbance units was reached. The culture was then split in 50 mL volumes, and the cells were pelleted by centrifugation at 4000 x g for 10 min. Cell pellets were stored at − 80 °C. The same protocol was performed in parallel for generating bacterial DNA that did not contain the ^15^N-isotope.

### Extraction of bacterial DNA

Cell pellets were vortexed and re-suspended in the remaining liquid. Three 50 mL Eppendorf tubes containing ^15^N-DNA were combined into one 50 mL Eppendorf tube and 25 mL of cell lysis solution was added. Next, 150 μL Proteinase K (20 mg/mL) were added followed by overnight incubation in the shaker at room temperature. A total of 7.5 mL of protein precipitation solution was added and vortexed for 20 s followed by incubation on ice for 10 min. The solution was then centrifuged (4000 x g for 10 min) and the remaining supernatant was divided evenly into two parts (~ 16.25 mL) and each were poured into clean Eppendorf tubes containing 17 mL cold isopropanol (IPA) to allow the DNA to precipitate. The precipitated DNA pellet was transferred in a clean silanized glass vial and subsequently washed using 3 mL 70% IPA and 3 mL 100% IPA. Pellets were air-dried and subsequently combined into one 50 mL Eppendorf tube.

The DNA was re-suspended in 10 mL 10 mM PIPES/5 mM MgCl_2_. A total of 150 μL RNAseA solution (4 mg/mL) was added followed by incubation at 37 °C for 2 h. A total of 5 mL protein precipitation solution was added followed by 20 s of vortexing, 5 min incubation on ice, and centrifugation for 10 min at 4000 x g. DNA precipitation was performed by addition of 2 mL cold IPA to each vial. The precipitated DNA was removed from the sample, placed in a clean, silanized glass vial, and washed twice with 1 mL 70% IPA and 1 mL 100% IPA. DNA pellets were air-dried and stored at − 20 °C.

### Reaction of calf thymus DNA (CT-DNA) or isotope-labeled bacterial DNA with DOX

DOX (100 μL, 0.6 mg/mL) in Tris-HCl buffer (10 mM, pH 7.4) was added to a reaction mixture containing formaldehyde (500 μL, 300 μM) in water and either CT-DNA (400 μL, 2.5 mg/mL), ^14^N-bacterial DNA (500 μL, 1 mg/mL) or ^15^N-bacterial DNA (500 μL, 0.8 mg/mL) in Tris-HCl buffer (10 mM, pH 7.4). The reaction mixtures were incubated at 37 °C for 24 h. The same reaction mixtures without DOX were used as negative controls. Isolation of DNA was performed by IPA precipitation. Briefly, 2 mL cold IPA were added to each vial. The precipitated DNA was removed from the sample, placed in a clean, silanized glass vial, and washed twice with 1 mL 70% IPA and 1 mL 100% IPA. The DNA pellet was dried under a nitrogen stream. All of the steps of this procedure were performed in silanized glass vials.

### Animal ethics

All procedures involving live vertebrates, including both mouse and canine patients, were reviewed and approved by the Institutional Animal Care and Use Committee (IACUC) at the University of Minnesota and were carried out in accordance with relevant guidelines and regulations. The IACUC protocols for the rodent study were 1807-36187A and 2006A38206, and the IACUC protocol for the canine patients was 1702-34548A. Additionally, all animal studies, both murine and canine, were performed in compliance with the Animal Research: Reporting of In Vivo Experiments (ARRIVE) guidelines [44].

### Mouse treatment

#### Single dose

Adult male C57BL/6 J mice (*n* = 6) were administered with a 10 mg/kg intraperitoneal injection of DOX or sterile saline vehicle. This dose was selected upon literature evaluation of similar studies involving an acute administration of DOX [45–48]. Mice were then sacrificed 24, 48, or 96 h following DOX injection (*n* = 2/time point). Control mice (*n* = 2) were sacrificed 48 h following vehicle injection. The liver and blood were harvested and stored at − 80 °C.

#### Weekly dose

Five week old male C57BL/6 N mice (*n* = 3/group) were administered once a week with DOX 4 mg/kg/week or equivalent volume of sterile saline vehicle by intraperitoneal injection for 3 weeks as we previously reported [49]. Animals were sacrificed at designated time points (1 or 3 weeks) after the last injection. Liver and blood samples were collected and stored at − 80 °C.

### Isolation of DNA from liver tissue samples

Genomic DNA from mice exposed to DOX was extracted with the QIAGEN Gentra Puregene Tissue Kit (Qiagen Sciences) following the manufacturer’s instructions with minor modifications. In brief, frozen liver tissues (270–390 mg) were minced with a razor blade while on dry ice. The minced tissues were lysed with 3 mL cell lysis solution and incubated for 5 min on ice to allow for degradation. The tissue was then homogenized using a tissue homogenizer set at low-medium speed for no more than 1 min. Additional 3 mL of cell lysis solution were added and mixed by inverting 25 times. Next, 30 μl of Proteinase K (20 mg/mL) were added and tubes were mixed by inverting 25 times and incubated overnight in a shaker at room temperature. A total of 30 μl RNase A solution (4 mg/mL) was added to each lysate and mixed before incubation for 2 h in a shaker at room temperature. Then, 2 mL of protein precipitation solution were added and tubes were vortexed vigorously for 20 s prior to centrifugation (2500 x g for 15 min). Supernatants were added to cold IPA, and DNA was precipitated and washed as previously described, with the only difference being the DNA pellets were air-dried. The DNA pellets were stored at − 20 °C. The amounts described above were reduced by a factor of 4 when using 50 mg of liver tissue.

### Recruitment and sample collection from patients undergoing chemotherapy with doxorubicin

Dogs with spontaneously arising tumors of various histologies undergoing treatment with a DOX-based chemotherapy protocol at the University of Minnesota Veterinary Medical Center were recruited. Dogs eligible for enrollment had a constitutional clinical signs score of 0 or 1 according to the Eastern Cooperative Oncology Group performance scale [50], body weight ≥ 10 kg, and adequate hematologic, renal, and hepatic function. Following written informed consent of each dog owner, blood (6–10 mL, depending on dog’s size) was collected via routine venipuncture into a potassium EDTA tube 7 days post-treatment with doxorubicin when dogs returned for their post-chemotherapy CBC per routine protocol at our institution.

### Isolation of DNA from blood tissue samples

Genomic DNA was extracted with the QIAGEN Gentra Puregene Blood Kit following the manufacturer’s instructions for DNA Purification from Whole Blood with minor modifications. In brief, 3 mL of whole blood were lysed with 9 mL red blood cell (RBC) lysis solution and mixed by inverting 10 times followed by 5 min of incubation at room temperature. Next, the solution was centrifuged for 2 min at 2000 x g to pellet the white blood cells. The supernatant was then discarded leaving approximately 200 μL of residual liquid. The pellet was resuspended in the residual liquid by vortexing vigorously. A total of 3 mL of cell lysis solution was added and tubes were vortexed. 30 μl RNase A solution (4 mg/mL) was added to each lysate and mixed by inverting 25 times followed by 15 min of incubation at 37 °C, which was followed by 3 min of incubation on ice. Then, 1 mL of protein precipitation solution was added and the tubes were vortexed vigorously for 20 s prior to centrifugation (2000 x g for 5 min). Supernatants were added to cold IPA, and DNA was precipitated and washed as previously described, with the only difference being the DNA pellets were air-dried. The dried pellets were stored at − 20 °C. The amounts described above were reduced by a factor of 6 when using about 0.5 mL of whole blood.

### DNA clean-up, hydrolysis and sample enrichment

Prior to hydrolysis and adduct enrichment, purified DNA samples and mouse liver DNA from the acute treatment study were dissolved in 2 mL 10 mM Tris + 1 mM EDTA (pH 7.0). Then, 2 mL of chloroform/isoamyl alcohol (24:1, purified DNA samples) or phenol/chloroform/isoamyl alcohol (25:24:1, mouse liver DNA samples) was added and the solution was vortexed vigorously for 60 s followed by centrifugation (2000 x g for 10 min), and the upper layer was collected and transferred into a clean 5 mL Eppendorf tube. The extraction was performed twice. After the second extraction, 200 μl 5 M NaCl were added. DNA was precipitated using cold IPA as previously described. The dried pellets were stored at − 20 °C until further use. The extraction was performed in an attempt to remove leftover drug from the samples.

Prior to DNA hydrolysis, DNA was re-dissolved in a 10 mM Tris-HCl/5 mM MgCl_2_ buffer (pH 7.4) solution. Initial digestion of DNA was performed overnight at room temperature by addition of 124 U/mg DNA (CT-DNA and bacterial DNA) or 600 U/mg DNA (liver and blood DNA) DNase I (recombinant, from *Pichia pastoris*). Then, an additional 124 or 600 U/mg DNA, 6.6 mU/mg DNA (CT-DNA and bacterial DNA) or 20 mU/mg DNA (liver and blood DNA) phosphodiesterase I (type II, from *Crotalus adamanteus* venom) and 46 U/mg DNA (CT-DNA and bacterial DNA) or 240 U/mg DNA (liver and blood DNA) of alkaline phosphatase (recombinant, from *Pichia pastoris*) were added and samples were incubated at 37 °C for 70 min. and followed by overnight incubation at room temperature. Enzymes were removed by centrifugation using a Centrifree ultrafiltration device (MW cutoff of 30,000, Millipore Sigma) at 2000 x g for 45 min. A 10–15 μL aliquot was removed from each sample for dGuo quantitation.

Samples were desalted and enriched using a Strata-X solid phase extraction (SPE) cartridge (33 μm, 30 mg/1 ml, Phenomenex). Briefly, the cartridge was pre-conditioned and equilibrated with 3 mL CH_3_OH and 1 mL H_2_O. Samples were loaded, and the cartridge was washed with 3 mL H_2_O and 1 mL 10% CH_3_OH in H_2_O. The two eluting fractions collected were 1 mL 100% CH_3_OH and 1 mL CH_3_OH + 2% formic acid. The fractions were evaporated until dry and stored at − 20 °*C. prior* to LC-MS analysis, samples were reconstituted in 500 μL (CT-DNA), 250 μL (bacterial DNA) or 10 μL (liver and blood DNA) 5% CH_3_OH in LC-MS grade water. For the DNA samples extracted from mouse liver and dog blood, the two SPE fractions were pooled together prior to LC-MS analysis.

### dGuo quantitation by HPLC-UV analysis

Quantitation of dGuo was carried out on an UltiMate 3000 UHPLC System (Thermo Fisher Scientific) with a UV detector set at 254 nm. A 250 × 0.5 mm Luna C18 100A column (Phenomenex, Torrance, CA) at 40 °C was used with a flow rate of 15 μl/min and a gradient from 5 to 25% CH_3_OH in H_2_O over the course of 10 min followed by an increase to 95% CH_3_OH in 3 min and a hold at 95% CH_3_OH for 5 min. The column was re-equilibrated to initial conditions for 8 min.

### LC-MS parameters

Samples were injected onto an UltiMate 3000 RSLCnano UPLC (Thermo Fisher Scientific) system equipped with a 5 μL injection loop. Liquid chromatography (LC) separation was performed on a capillary column (75 μm ID, 20 cm length, 10 μm orifice) created by hand packing a commercially available fused-silica emitter (New Objective) with 5 μm Luna C18 bonded separation media (Phenomenex). Gradient conditions were 1000 nL/min for 5.5 min at 5% CH_3_CN in 0.05% formic acid aqueous solution, then decreased to 300 nL/min followed by a linear gradient of 1%/min over 44 min for the untargeted screening and over 30 min for the targeted MS/MS analysis. Column wash was performed with a flow rate of 300 nL/min at 98% CH_3_CN for 5 min (untargeted screening) or at 95% CH_3_CN for 2 min (targeted MS/MS analysis). Re-equilibration was performed with a flow rate of 1000 nL/min at 5% CH_3_CN for 5 min (untargeted screening) or for 1 min (targeted MS/MS analysis). The injection valve was switched at 5.5 min to remove the sample loop from the flow path during the gradient. All MS data was acquired on an Orbitrap Fusion Tribrid Mass Spectrometer (Thermo Fisher Scientific). Positive mode electrospray ionization and nanospray (300 nL/min) were used on a Thermo Scientific Nanoflex ion source with a source voltage of 2.2 kV, a capillary temperature of 300 °C, a S-Lens RF level set at 60%, and EASY-IC lock mass (*m/z* 202.0777) enabled.

### Constant neutral loss (CNL)-MS^n^ data-dependent acquisition (DDA)

CNL-MS^n^ DDA was performed by repeated full scan detection followed by MS^2^ acquisition and constant neutral loss triggering of MS^3^ fragmentation. Full scan (range 200–2000 Da) detection was performed by setting the Orbitrap detector at 60,000 resolution with 1 microscan, automatic gain control (AGC) target settings of 2.0E5, and maximum ion injection time set at 50 ms. The most intense full scan ions were fragmented over a 2 s cycle. The MS^2^ fragmentation parameters were as follows: quadrupole isolation window of 1.6, HCD collision energy of 20% ± 10%, Orbitrap detection at a resolution of 7500, AGC of 2.0E5, 1 microscan, maximum injection time of 50 ms, and EASY-IC lock mass (*m/z* 202.0777) enabled. Data-dependent conditions were as follows: triggering intensity threshold of 2.5E4, repeat count of 1, exclusion duration of 30 s, and exclusion mass width of ±5 ppm. The MS^3^ fragmentation parameters were as follows: HCD fragmentation, 2 amu isolation window, collision energy of 20% ± 10%, Orbitrap detection at a resolution of 7500 upon the observation of neutral losses (± 5 ppm) of 116.0474 (− dR), 151.0494 (− G), 135.0545 (− A), 126.0429 (− T), 111.0433 (− C), 156.0346 (− ^15^N-G), 140.0413 (− ^15^N-A), 128.037 (− ^15^N-T), or 114.0344 (− ^15^N-C) between the parent ion and one of the most intense product ions from the MS^2^ spectrum, provided minimum signal of 2.5E4, AGC of 2.0E5, maximum injection time of 50 s, and EASY-IC lock mass (*m/z* 202.0777) enabled.

### Targeted data acquisition

Targeted MS^2^ acquisition was performed with a quadrupole isolation window of *m/z* 1.5 centered on *m/z* 609.2, 598.2, 735.2, 592.1, 619.2, 608.1, 743.1, 358.1, 376.1, 378.6, 470.2, 472.2, 363.1, 381.1, and 383.6 during the time span 0–14 min, *m/z* 541.2, 345.2, 356.2, 373.2, and 432.2 during the time span 12–20 min, *m/z* 415.2, 531.2, 340.2, and 425.1 during the time span 14–20 min, *m/z* 680.2, 809.3, 814.2, 685.2, 546.2, 544.2, and 791.9 during the time span 20–41 min, and *m/z* 823.3 during the time span 0–41 min. The other settings were: scan range 80–1000, HCD fragmentation of 20% ± 10%, Orbitrap detection at a resolution of 60,000, AGC of 5.0E4, 1 microscan, maximum injection time of 118 ms, RF lens set at 60% and EASY-IC lock mass (*m/z* 202.0777) enabled.

## Supplementary Information

**Additional file 1: Table S1**. Canine cancer patient information.

## Data Availability

The datasets used and analyzed in the current study are available from the corresponding author upon reasonable request.

## Abbreviations

A: Adenine
AH_2_O: Adenine and Water (when referring to a neutral loss)
C: Cytosine
CBC: Complete Blood Count
CH_3_CN: Acetonitrile
CH_3_OH: Methanol
CHOP: Cyclophosphamide, Hydroxydaunorubicin (doxorubicin), Oncovin (vincristine), and Prednisone
cMCT: Cutaneous Mast Cell Tumor
CNL: Constant Neutral Loss
CT-DNA: Calf Thymus DNA
dG: Deoxyguanosine
DOX: Doxorubicin
DOXol: Doxorubicinol
dR: 2′-Deoxyribose
EASY-IC: Easy Internal Calibration
EDTA: Ethylenediaminetetraacetic acid
EIC: Extracted Ion Chromatogram
FS: Full Scan
G: Guanine
GH_2_O: Guanine and Water (when referring to a neutral loss)
h: Hour(s)
HRAM: High-resolution/Accurate-mass
IE: Iterative Exclusion
IPA: Isopropanol
LC: Liquid Chromatography
LLOQ: Lower Limit of Quantitation
LOD: Limit of Detection
min: Minute(s)
MOA: Mechanism of Action
MS: Mass Spectrometry
ND: Not Detected
NL: Neutral Loss
RBC: Red Blood Cell
RT: Retention Time
s: Second(s)
SPE: Solid Phase Extraction
T: Thymine
U: Units
UW: University of Wisconsin
