# Supplementary material for: Identification of new candidate biomarkers to support doxorubicin treatments in canine cancer patients
Source: BMC Vet Res. 2021 Dec 7;17:378. doi: 10.1186/s12917-021-03062-x (PMC8650425; doi:10.1186/s12917-021-03062-x)
Supplement: Supplementary file 1 — Additional file 1: Table S1. Canine cancer patient information. [file 12917_2021_3062_MOESM1_ESM.docx]

**Table S1.** Canine cancer patient information.

|  | **Dog 1** | **Dog 2** | **Dog 3** |
| --- | --- | --- | --- |
| **Signalment** | 6 year old female spayed Irish Setter | 12 year old male neutered German Shepherd Dog | 9 year old male neutered Cocker Spaniel |
| **Cancer type** | High grade lymphoma | High grade lymphoma | High grade lymphoma |
| **Chemotherapy protocol** | UW-Madison CHOP-19 | Modified UW-Madison CHOP-19 | UW-Madison CHOP-19 |
| **Number of doxorubicin treatments at sample collection** | 3 | 5 | 4 |
| **Doxorubicin dose (mg/m^2^)** | 29.9 | 29.6 | 29.8 |
| **Doxorubicin dose (mg/kg)** | 0.9 | 0.9 | 1.4 |
